# Understanding Park Golf Participation Among Older Adults: The Role of Social Support in Health Behavior Through the Lens of the Theory of Planned Behavior

**DOI:** 10.3390/bs14111062

**Published:** 2024-11-07

**Authors:** Dohun Kim, Yunduk Jeong

**Affiliations:** 1 Department of Sports and Welfare, Korea National University of Transportation, Chungju 27469, Republic of Korea; kpga0000@hanmail.net; 2 College of General Education, Kookmin University, Seoul 02707, Republic of Korea

**Keywords:** park golf, older adults, theory of planned behavior, event image, social support

## Abstract

Addressing gaps in the existing research, this study investigates how event image affects attitudes, subjective norms, and perceived behavioral control (PBC); how attitudes, subjective norms, and PBC impact behavioral intention, applying the theory of planned behavior (TPB) framework; and how social support moderates these variables among older participants in park golf. We gathered data from senior park golf tournament participants and utilized confirmatory factor analysis to validate the measurement scale, assessing factor loadings, average variance extracted (AVE), and construct reliability (CR), while our analysis of Cronbach’s alpha ensured scale reliability. We also employed structural equation modeling with maximum likelihood estimation to analyze the positive relationships and conducted a moderation analysis using Jamovi software. The results indicate the significant effect of event image on attitudes, subjective norms, and PBC, as well as the significant effect of attitudes, subjective norms, and PBC on behavioral intention. Moreover, social support moderates the relationship between subjective norms and behavioral intention.

## 1. Introduction

Numerous societies are confronted with an increasing number of elderly individuals, with predictions that by 2050, approximately one in six individuals will be 60 years of age or older [1]. As individuals age, they may encounter challenges in participating in routine activities with their families and communities due to changes in their social environments and declines in their physical and mental health [2]. Actively engaging in leisure activities can be a valuable method of promoting social connections and overall well-being among older individuals, as well as mitigating the effects of aging on both physical and mental health, despite these challenges. In addition to enhancing physical health, engaging in leisure activities in old age also contributes to cognitive engagement, emotional equilibrium, and a sense of accomplishment among senior adults. Furthermore, participation in these activities fosters social connections, thereby establishing meaningful support systems and, as a result, improving the quality of life for seniors [3].

Physical activity is one of the many different types of leisure that older individuals enjoy, and it has long been shown to be beneficial. According to guidelines for physical activity, people 65 years of age and older should partake in a variety of recreational activities that include aerobic workouts, muscle strengthening, and balancing training [4]. A growing body of evidence suggests that physical activity has many positive health effects, including the management and prevention of a number of chronic illnesses [5]. Research has also shown that physical activity improves older people’s cognitive health [6]. Furthermore, regular physical activity has a number of positive effects on mental health in older persons. These benefits include lowering symptoms of anxiety and depression, boosting memory and cognitive performance, and improving the quality of sleep. Moreover, leading an active lifestyle fosters a sense of accomplishment and purpose, which enhances happiness and contentment as one matures.

Park golf has become a well-liked and easily accessible choice as older folks’ interest in a variety of leisure activities grows [7]. Park golf, sometimes referred to as small golf, is a relatively new concept that combines the words “golf” and “park”. Originating in Hokkaido, Japan, it has become popular as a preferred leisure activity for seniors all around the world, including Korea, Australia, China, and Hawaii. Park golf was once thought of as a senior citizen sport, but it is now seen as a fun family pastime that can be played in urban parks by all ages. Park golf is a great way for seniors to be active and engaged in their communities because it provides a special combination of physical exertion, social connection, and leisurely enjoyment [8]. Long-term maintenance of a healthy lifestyle requires addressing environmental, psychological, physical, and social variables. It is predicted that park golf will become more popular in the future among the many physical activities that support a healthy lifestyle. Thus, investigating the antecedent factors influencing the decision to keep playing park golf can help the senior sports and park golf sectors to flourish.

This study applies the theory of planned behavior (TPB) to examine the factors influencing the intention to continue playing park golf. Over the past few decades, the theory of planned behavior, which is an expansion of the theory of reasoned action (TRA), has gained popularity as a paradigm for comprehending human behavior in a variety of academic fields [9,10]. According to this theory, a person’s intentions to engage in a behavior are influenced by their attitudes toward that action, subjective norms, and perceived behavioral control (PBC). A stronger intention is associated with a higher likelihood of carrying out the actual behavior. Additionally, previous studies have shown that attitudes, subjective norms, and PBC can be influenced by image [11]. Similar to golf, park golf is a year-round sport, and as such, perceptions of park golf tournaments can have a good impact on attitudes, subjective norms, and PBC related to park golf.

To set itself apart from earlier studies, this study also investigates the moderating role of social support. Given that they frequently get help from others around them, older people who have a lot of social support are more likely to keep positive attitudes and behaviors when participating in everyday activities or physical activity [12]. It is also worthwhile to investigate how attitudes, subjective norms, and PBC influence behavioral intention and how social support acts as a moderator on these pathways. By using the TPB, the current study fills in several gaps in the literature by investigating three key areas: (1) the impact of event image on attitudes, subjective norms, and PBC; (2) the relationship between behavioral intention, attitudes, and PBC; and (3) the moderating effects of social support on the variables of interest.

## 2. Theoretical Background

### 2.1. The Theory of Reasoned Action (TRA) and Theory of Planned Behavior (TPB)

Fishbein and Ajzen [13] contended that human cognition is predominantly rational, with behaviors primarily arising from logical assessments and calculations aimed at optimizing individual interests. Expanding on this premise, Fishbein and Ajzen [13] developed the TRA to elucidate and forecast economic and social phenomena. The TRA is based on the idea that individuals form intentions to perform behaviors based on their attitudes, subjective norms, and beliefs about the outcomes of those behaviors. The theory does not assume that behavior is always rational but rather provides a framework for predicting how individuals plan or justify their actions based on their expectations of outcomes. According to the TRA, individuals anticipate that their actions will lead to certain results and behave accordingly [14].

The TRA identifies attitudes and subjective norms as variables that comprise rational behavior. Attitudes are a significant notion across multiple disciplines, including social psychology, marketing, and education, facilitating the comprehension and anticipation of human psychology and behavior. Ajzen [15] defined attitude as “the degree to which a person has a favorable or unfavorable evaluation or appraisal of the behavior in question”. This means that attitudes are based on how positively or negatively individuals perceive a particular behavior. For example, if an individual believes that playing park golf is enjoyable and beneficial, this would constitute a positive attitude towards the behavior. In the TRA and TPB frameworks, attitudes are treated as a single evaluative dimension, rather than being broken down into cognitive, affective, or behavioral components, as the theories aim to predict behavioral intentions based on overall evaluations of the behavior. Of the three TPB variables, attitudes exert the most significant influence on behavioral intention, and since attitudes are resistant to change once established, understanding attitudes is crucial for predicting behavioral intention [16].

Subjective norms are a person’s interpretation of social pressures and other people’s expectations. They include, for instance, how much a person believes that friends, family, mentors, coworkers, or relatives approve or disapprove of a certain action [15]. Since humans are social animals, it is very likely that people will engage in actual actions when dealing with others because they want to live up to their expectations [10]. Stated differently, people are more inclined to choose to engage in a behavior if they believe that others around them have positive opinions about it; on the other hand, people are more likely to choose not to engage in the behavior if they believe that others around them have negative opinions about it [13]. Subjective norms are shaped by outside factors, whereas attitudes are shaped by personal experiences. This is how attitudes and subjective norms differ from one another.

After its inception, the TRA was extensively utilized by numerous researchers until the late 1990s, when specific limitations were identified. Participants in park golf may have good attitudes toward the activity, and their family and friends may likewise endorse park golfing. Nonetheless, limitations such as physical injuries or personal obligations may hinder persons from participating in park golf. Such situations involve behavioral elements that individuals may struggle to regulate, which can affect their behavioral intentions [10]. Thus, Ajzen [15] introduced the TPB to address the shortcomings of the TRA by integrating PBC. This hypothesis illustrates individuals’ perceptions regarding the simplicity or complexity of executing a behavior autonomously. PBC exhibits numerous parallels with self-efficacy, since both pertain to individuals’ views of their capacity to surmount challenges. A nuanced distinction occurs in that perceived behavioral control includes both internal and external control variables, while self-efficacy is only founded on internal control components [17].

### 2.2. Event Image

An event image is defined as “the cumulative interpretation of meanings or associations attributed to events by consumers”, as noted by Gwinner [18]. An individual’s interpretation of a particular event can be influenced by various factors, including the event type (e.g., sports, arts festivals, music concerts, charity events, networking events, and product launches), event attributes (such as history, professional standing, venue, scale, distinctiveness, perishability, and labor intensity), and personal factors (including prior experiences with events, event knowledge and education, sense of agency, values, personality traits, self-construal, age, objectives, and cognitive biases) [18]. Furthermore, the event image may differ based on whether the individual is a spectator or a participant in the sporting event [19]. For example, viewers who passively participate in an event by occupying the stands often emphasize the social and historical dimensions of the occasion. In contrast, people who engage actively in the event may experience a more profound connection between their emotions and the event’s physical elements. Kaplanidou and Vogt [20] established the idea of a Sport Event Image (SEI), consisting of 13 components, to evaluate a wide range of event pictures. This study concentrated on the emotion-related components of the SEI, including sensations of boredom versus excitement, melancholy versus joy, and gloominess versus cheerfulness. This emphasis corresponds with our previous discussion indicating that participants often closely link emotions to their event experiences.

### 2.3. Social Support

Social support encompasses both instrumental and emotional dimensions and is defined as interpersonal interactions that involve positive emotional expression, affirmation of an individual’s values and beliefs, and provision of practical aid or assistance when required [21,22]. The term “instrumental support” describes the exchange of useful help for routine tasks or emergencies. According to Rodrigues et al. [23], this assistance includes not just monetary contributions but also tangible goods and services like providing care or helping with transportation. There are several methods to provide instrumental support to senior citizens playing park golf, such as giving them golf clubs and equipment, conducting coaching sessions, and making travel arrangements to guarantee their participation. Furthermore, setting up events and finding appropriate venues are essential ways to offer instrumental support. Conversely, emotional support entails people discussing their feelings, showing empathy, and receiving validation from one another. According to Rodrigues et al. [23], this comprises social interaction components like talking about one’s feelings and personal concerns as well as giving advice on them. For older people playing park golf, emotional support requires a caring and compassionate environment; this means paying attention to their concerns, giving them words of encouragement, and encouraging them to form friendships and a sense of community. Furthermore, providing comfort and empathy in trying times is essential for emotional support.

## 3. Theoretical Research Hypotheses Development

Previous studies have reported that image can influence attitudes, subjective norms, and PBC. For example, applying the TPB, Jeong and Kim [24] analyzed the behavioral intentions of water-sports tourists and revealed that destination image significantly influences attitudes, subjective norms, and PBC. Similarly, Alenezi et al. [25] found a significant relationship between image, subjective norms, and self-identity in relation to e-learning acceptance among Saudi university students. Meanwhile, Jalilvand et al. [26] explored the relationship between destination image, attitudes, and intention to travel to Iran, demonstrating that destination image is a precursor to raising attitudes. Therefore, building on these previous studies, this study proposes the following hypotheses:

**H1-1:**  *Event image positively influences attitudes.*

**H1-2:**  *Event image positively influences subjective norms.*

**H1-3.**  *Event image positively influences PBC.*

Existing studies consistently demonstrate that attitudes, subjective norms, and PBC are key in generating behavioral intention. For example, using an extended version of the TPB, Liu et al. [27] analyzed the intention of older individuals to engage in walking behavior and demonstrated that attitudes and PBC are significantly linked to the behavioral intention of older adults. Similarly, Seonwoo and Jeong [28] examined the structural relationships between mentoring, attitudes, subjective norms, PBC, and intentions toward career pursuit and found positive influences of attitudes, subjective norms, and PBC on intentions. Si et al. [29] investigated the factors influencing users’ intention and behavior toward sustainable usage by integrating them into the TPB framework and revealed that attitudes, subjective norms, and PBC exert significant positive effects on sustainable usage intention. Hence, these observations contribute to the following hypotheses:

**H2-1:**  *Attitudes positively influence behavioral intention.*

**H2-2:**  *Subjective norms positively influence behavioral intention.*

**H2-3:**  *PBC positively influences behavioral intention.*

Concerning the moderating influence of social support on the relationships between attitudes and behavioral intention, subjective norms and behavioral intention, and PBC and behavioral intention, as previously addressed, attitudes, subjective norms, and PBC are expected to positively influence behavioral intention. Previous studies have also suggested that social support is pivotal in generating intention. For example, Jeong and Kim [30] explored the effect of social support on intention to continue exercise and found that all sub-factors of social support have a positive effect on intention. On the other hand, little research has investigated the relationships between social support and attitudes, subjective norms, and PBC. Hence, we propose the following hypotheses:

**H3-1:**  *Social support moderates the influence of attitudes on behavioral intention.*

**H3-2:**  *Social support moderates the influence of subjective norms on behavioral intention.*

**H3-3:**  *Social support moderates the influence of PBC on behavioral intention.*

**Figure 1:**
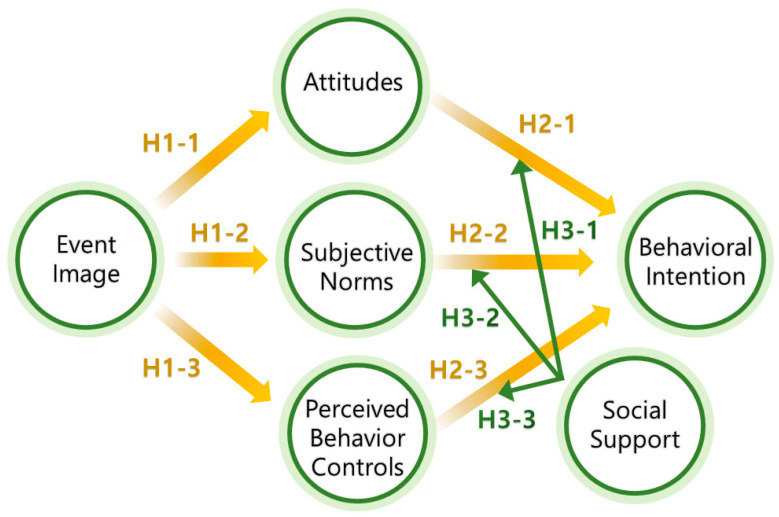
Proposed conceptual model. Yellow represents paths exploring direct positive effects between variables, while green indicates paths exploring the moderating effects.

After reviewing the available literature, we utilized the conceptual framework depicted in Figure 1.

## 4. Method

### 4.1. Design

This study employs a cross-sectional design, utilizing survey data collected from senior park golf participants in South Korea to analyze factors influencing their behavioral intentions toward park golf participation.

### 4.2. Setting

The study was conducted at six senior park golf tournaments held across South Korea between May and July 2023. These tournaments provided a suitable setting for targeting a population of amateur senior park golf players.

### 4.3. Participants

We targeted amateur park golf athletes aged 60 and older who attended six senior park golf tournaments in South Korea. Participation was voluntary, and individuals were included only if they willingly consented after a full explanation of the study’s objectives and procedures. We employed a convenience sampling approach, distributing a total of 268 questionnaires and collecting 252 valid responses for analysis. As a non-random sampling method, convenience sampling may limit the generalizability of the findings, as it does not guarantee a representative sample of the entire population of senior park golf athletes. Demographic characteristics were recorded, including gender (73.9% male, 26.1% female), income levels (20.8% less than USD 20,900, 38.9% between USD 20,901 and USD 41,800, 22.1% between USD 41,801 and USD 62,700, and 18.2% above USD 62,701), and educational background (44.2% university graduates, 26.7% with an associate degree, 19.1% high school graduates, and 9.9% with a graduate degree).

### 4.4. Measure

The survey comprised inquiries pertaining to event image, attitudes, subjective norms, PBC, behavioral intention, and social support. All the measurement scales utilized in this study were sourced from well-established academic sources and had previously undergone thorough validation in existing studies. We assessed event image using three items adapted from Kaplanidou and Vogt [20]; attitudes using three items adapted from Ajzen [31] and Seonwoo and Jeong [28]; subjective norms using three items from Ajzen [31] and Yu and Jeong [10]; PBC using three items adopted from Ajzen [15] and Perugini and Bagozzi [32]; behavioral intention using three items from Seonwoo and Jeong [28] and Yu and Jeong [10]; and social support using three items adopted from Park [33].

Participants utilized a 5-point Likert scale ranging from 1 (strongly disagree) to 5 (strongly agree) to provide their responses. The study instruments were assessed for content validity by a panel comprising two experts in the sociology of sports. These panelists evaluated the items for each construct, considering aspects such as relevance, representativeness, and clarity. Incorporating their valuable feedback, we refined the initial questionnaire, which culminated in the final version employed for distribution. Furthermore, prior to administering the survey, approval was sought and obtained from the university’s Institutional Review Board.

### 4.5. Data Analysis

Data collected from the questionnaire were computed using the SPSS version 25 and AMOS version 24 software packages. SPSS was used to conduct the frequency, correlation, and reliability analyses. AMOS was used to perform confirmatory factor analysis and structural equation modeling (SEM). Jamovi version 2.3 was used to conduct moderating effects.

### 4.6. Validity and Reliability

In this study, we employed confirmatory factor analysis (CFA) with maximum likelihood estimation in AMOS to validate the dimensional structure of the measurement model. The goodness-of-fit indices for the CFA all fell within the recommended thresholds (χ²/*df* = 2.635, NFI = 0.942, RFI = 0.926, IFI = 0.963, TLI = 0.953, CFI = 0.963, and RMSEA = 0.074), as outlined by Hooper et al. [34]. To establish convergent validity, we calculated the factor loadings, construct reliability (CR), and average variance extracted (AVE). All factor loadings exceeded the recommended threshold of 0.50, CR values ranged from 0.939 to 0.953, and AVE values ranged from 0.837 to 0.872, meeting the criteria suggested by Hair et al. [35] and Tabachnick and Fidell [36]. Table 1 presents these results in detail, confirming satisfactory convergent validity and reliability, with Cronbach’s alpha values also exceeding the 0.7 threshold.

Discriminant validity was assessed by comparing the square roots of AVE with the correlation coefficients between constructs. In Table 2, the diagonal elements (in bold) represent the square roots of the AVE values for each construct. All diagonal elements were greater than the off-diagonal elements, demonstrating satisfactory discriminant validity.

The reliability assessments, measured using Cronbach’s alpha for the constructs of event image, attitudes, subjective norms, PBC, behavioral intention, and social support (ranging from 0.891 to 0.939), all exceeded the recommended threshold of 0.7, confirming the robustness of the measurement instruments in accordance with the guidelines outlined by Fornell and Larcker [37] (see Table 1 for details).

## 5. Results

### 5.1. Model Fit and Structural Model

**Figure 2:**
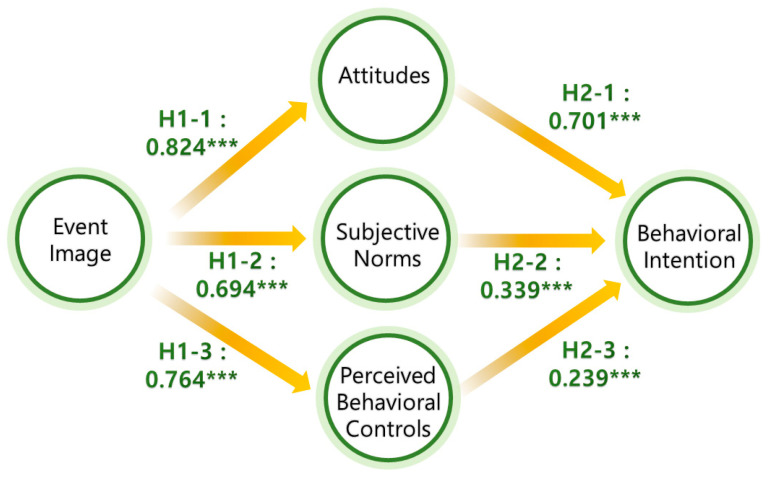
Structural model results. *** *p* < 0.001.

We tested the proposed relationships using SEM, and all fit indices indicated a well-fitting model (χ²/*df* = 2.604, CFI = 0.934, RMSEA = 0.073). The analysis confirmed significant positive relationships between event image and attitudes (*β* = 0.824, *p* < 0.001), subjective norms (*β* = 0.694, *p* < 0.001), and PBC (*β* = 0.764, *p* < 0.001). Similarly, attitudes (*β* = 0.701, *p* < 0.001), subjective norms (*β* = 0.339, *p* < 0.001), and PBC (*β* = 0.239, *p* < 0.001) all significantly influenced behavioral intention. See Figure 2 for a detailed presentation of the results.

### 5.2. Tests of Moderating Effect

The moderation analysis revealed that social support significantly moderated the relationship between subjective norms and behavioral intention (*β* = 0.172, *p* = 0.036), indicating that higher social support strengthens this relationship. However, the interaction between social support and attitudes, as well as PBC, did not yield significant results (*p* > 0.05). Table 3 highlights the key moderation effects and their significance levels for clarity.

## 6. Discussion and Conclusions

### 6.1. Theoretical Implications

In the context of park golf, the current study not only highlights the beneficial effects of event image in forming attitudes, subjective norms, and PBC (H1.1, 1.2, and 1.3), but it also presents new insights that improve our knowledge of participant behavior and event image in the field of event management. Furthermore, the results of our study support the validity of our findings and are consistent with previous studies [24,25,26]. The capacity of park golf events to elicit pleasant associations and views among participants can be linked to the mechanisms via which they alter attitudes, subjective norms, and PBC. Park golf tournaments initially draw participants in due to their prestige and excitement, which creates a feeling of anticipation and desire to participate, which in turn creates a positive attitude toward being involved. This positive attitude is crucial because it directly influences participants’ decisions to continue engaging in the activity, as suggested by the TPB. Second, the favorable perception of the competition affects participants, making them feel under social pressure to conform to societal standards and expectations. The stronger the social pressure, the more participants feel compelled to follow through on their intention to participate, reinforcing the importance of subjective norms. This, in turn, influences participants’ propensity to play park golf. Finally, the favorable representation of the competition boosts players’ confidence in their abilities and proficiency to play park golf, strengthening their sense of control over their behavior and enhancing their incentive to take part in the activity. PBC is essential, as it empowers individuals by making them feel capable of successfully performing the behavior, thus increasing their likelihood of participation.

This study investigates the correlation between event image and participant behavior in event management, specifically within the realm of park golf, an area that has been inadequately explored in the current literature. By revealing these previously unexamined features, we augment the academic understanding of park golf and event management, hence facilitating future study opportunities. Our findings emphasize the significance of event image and provide critical insights for enhancing participant behavior in the planning and management of park golf events. Moreover, our research provides actionable insights for event planners in the park golf industry, suggesting strategies to enhance event perception and boost participant engagement. By comprehending the complex relationship between event image and participant conduct, professionals can tailor their management tactics to enhance the effectiveness and success of park golf tournaments.

This study demonstrates that attitudes, subjective norms, and perceived behavioral control strongly influence behavioral intention (H2.1, 2.2, and 2.3). These findings correspond with previous research indicating that attitudes, subjective norms, and perceived behavioral control enhance behavioral intention [27,28,29]. The methods by which attitudes, subjective norms, and perceived behavioral control affect the behavioral intentions of older park golf participants are as follows: Older adults who possess favorable perceptions of park golf, considering it fun, soothing, and health-promoting, are more inclined to cultivate a robust intention to participate in this sport. These positive attitudes frequently arise from individual experiences, societal factors, and the supposed health advantages linked to park golf. Attitudes are crucial as they serve as the individual’s evaluation of the behavior, and positive attitudes typically lead to stronger intentions to participate. Additionally, the subjective norms of elder participants include the views, anticipations, and support from friends, family, or classmates concerning involvement in this recreational activity. Older folks are more willing and driven to participate in park golf when they perceive support or endorsement from significant others. The perceived social endorsement strengthens subjective norms, making individuals more likely to engage in park golf to meet these external expectations. Consequently, subjective norms influence individuals’ behavioral intentions about park golf. Moreover, PBC encompasses individuals’ self-assurance in their competencies, expertise, resources, and the contextual elements affecting their capacity to engage in this activity. The higher the perception of control, the more confident individuals feel in their ability to engage in park golf, which directly strengthens their intention to participate. Increased perceptions of control about participation in park golf, including feelings of competence, access to essential equipment, and the availability of convenient playing places, can enhance a person’s intents to partake in this leisure activity. Individuals who perceive themselves as having sufficient control over their participation in park golf are more inclined to have stronger intentions to engage in the activity.

This study reveals that attitudes are the most significant component in shaping behavioral intention, contrary to recent studies that identified subjective norms or perceived behavioral control as the primary influences. Yu and Jeong [10] utilized the theory of planned behavior to investigate the structural links among attitudes, subjective norms, perceived behavioral control, and career pursuit intention in aspiring e-sport athletes, establishing that subjective norms are the predominant factor enhancing intention. In contrast, Liu et al. (2022) [27] examined the factors influencing older individuals’ intention to walk in their area, revealing that perceived behavioral control (PBC) is the most critical determinant of this intention. The present analysis verifies that attitudes have the greatest effect size, consistent with previous studies [29,38]. These findings underscore the necessity for a sophisticated comprehension of the elements affecting behavioral intention, indicating that attitudes may assume a more critical role than previously believed. Attitudes, as personal evaluations, shape how individuals approach behavior, and in this study, they were found to be a more dominant predictor than external social pressures or perceived control. Attitudes should be regarded as a fundamental factor in the formulation of interventions designed to promote desirable behaviors.

Furthermore, this study makes a substantial theoretical contribution by looking at social support as a moderating element. The manner in which social support moderates the associations between behavioral intention and subjective norms has not been explored in previous research. The main conclusions of this study, however, show that social support has an amplifying effect and does, in fact, regulate these associations. The ability of social support to mold attitudes and influence behavior within the park golf community is the fundamental cause of its moderating effect on the relationship between subjective norms and the intention to continue park golf among older participants. Social support plays a crucial role in how people understand and respond to subjective norms, which include the expectations and ideas that people hold about playing park golf that are held by important people like friends, family, and peers. Furthermore, social support provides people with affirmation, encouragement, and endorsement of their park golfing activities, which influences how they perceive and react to arbitrary norms. Older participants are more likely to feel driven or obligated to conform their behavior to these perceived standards and expectations when they observe strong social support for park golf involvement from their social networks. As such, this encouragement fortifies their resolve to continue playing park golf for an extended amount of time.

Although social support significantly moderated the relationship between subjective norms and behavioral intention, its moderating effect on attitudes and PBC was not significant. This suggests that while social support strengthens social pressures (subjective norms), it does not necessarily amplify personal attitudes or perceived control over the behavior. These results indicate that while external social support plays a significant role in strengthening the influence of social pressures (subjective norms), participants’ own attitudes and confidence in their ability to perform the behavior (PBC) remain the primary factors driving their behavioral intentions, independent of social support.

While the traditional TPB typically incorporates instrumental support into PBC and emotional support into subjective norms, this study argues that social support, particularly among older adults, operates as a distinct construct that influences behavioral intention beyond these two dimensions. Social support provides both practical assistance and emotional encouragement, which together create a broader, multifaceted impact that cannot be fully captured by PBC or subjective norms alone. This is especially relevant in the context of park golf, where older participants may rely heavily on social networks for motivation, assistance, and validation. Therefore, treating social support as a distinct moderating factor allows for a deeper understanding of its amplifying effects on behavioral intention.

Overall, this study highlights the pivotal role of social support as a moderating factor in shaping the relationships between subjective norms, behavioral intention, and park golf participation among older individuals. Therefore, understanding the interplay between social support, subjective norms, and behavioral intention is crucial for designing effective interventions aimed at promoting and sustaining park golf participation among older individuals, which ultimately enhances their overall well-being and social connectedness within the community.

### 6.2. Practical Implications

Based on the results of our study, we suggest a number of tactics to enhance older players’ opinions of park golf tournaments. (1) Increasing prestige and excitement: Increasing the prestige and excitement associated with park golf competitions can draw attention from participants and generate a sense of eagerness and expectation for taking part. This can be achieved by creatively structuring the events, inviting esteemed speakers, or holding unique award ceremonies that highlight the tournament’s importance. (2) Fostering positive social norms: Creating a positive impression of the competition may persuade attendees to live up to expectations and perceived norms. Fostering a sense of belonging and encouraging more participation can be achieved through establishing welcoming, encouraging environments where people are treated with respect and feel appreciated. (3) Increasing participant confidence: Giving participants a favorable impression of the competition can help them feel more confident about their capacity to play park golf. Facilitating skill-building seminars, individual coaching sessions, or showcasing the accomplishments of more senior participants can boost self-esteem and reaffirm the desire to engage fully in the event.

Furthermore, based on our study’s findings, we suggest the following strategies to enhance attitudes, subjective norms, and PBC among senior park golf players. (1) Increasing positive attitudes: Stress the fun, relaxation, and health benefits of park golf to older participants in order to help them develop positive attitudes toward the game. Positive attitudes toward park golf can be developed by offering chances for customized experiences, social connections, and educational sessions about the game’s health benefits. (2) Fostering favorable subjective norms: Establish a welcoming environment where senior players get support, affirmation, and encouragement from friends, family, and peers for playing park golf. Organizing park golf-related social activities, neighborhood get-togethers, and support systems can promote a feeling of community and good social impacts. (3) Strengthening PBC: By addressing variables that affect older people’s perceived control, we can provide them with the self-assurance and tools they need to play park golf. This may be giving out reasonably priced equipment, holding workshops to improve skills, making park golf facilities easily accessible, and removing any environmental barriers that would prevent people from participating.

Finally, based on our findings, we propose various strategies to enhance social support for older adults participating in park golf. (1) Fostering community networks: Promote the development of supportive networks within the park golf community by organizing social meetings, collaborative activities, and communal events. These events offer elder participants the chance to forge contacts, share thoughts, and provide reciprocal encouragement and support. (2) Providing educational materials: Facilitate access to instructional resources and workshops that emphasize the benefits of park golf participation for seniors, encompassing its physical, mental, and social advantages. Distributing information about the beneficial effects associated with park golf can strengthen participants’ beliefs and motivations. (3) Fostering peer support: Establish peer support circles or buddy networks that enable older participants to develop connections and provide mutual assistance during their park golf experience. Motivating individuals to share their experiences, provide information, and offer emotional support can strengthen social connections and enhance overall happiness with involvement. (4) Recognizing and celebrating milestones: Acknowledge and celebrate the milestones and achievements of senior park golf participants, whether they signify skill enhancement, personal victories, or unwavering commitment to the sport. Commemorating their efforts and accomplishments can foster a sense of connection and affirmation within the park golf community.

### 6.3. Limitation and Future Research

The present study possesses multiple limitations that subsequent research may rectify. Firstly, this study exclusively examines park golf participants in East Asia, perhaps restricting the generalizability of its findings to participants in other global locations. Consequently, subsequent researchers ought to validate the proposed model across other regions, including North America and Western Europe. Secondly, future studies may incorporate participants’ personality features (e.g., the Big Five personality traits and attachment styles) as control variables to furnish park golf administrators with essential insights into participant behaviors influenced by individual characteristics. Moreover, examining the interaction effects of personality traits and other variables may provide a more profound comprehension of participant behavior. Thirdly, to comprehensively comprehend park golf player behavior, supplementary aspects that may affect behavioral intention must be taken into account. Qualitative research or mixed-method techniques may elucidate the intricate interaction of factors affecting behavioral intention. Fourthly, the use of a convenience sampling method in this study may limit the generalizability of the findings. Since participants were not randomly selected, the results may not fully represent the broader population of senior park golf athletes. Future research should consider employing random sampling methods to enhance the representativeness of the sample and provide more generalizable insights. Finally, although the present study employs social support as a moderating component, future research could investigate further moderating variables to augment the model’s comprehensiveness.

### 6.4. Conclusions

This study sheds light on the factors influencing park golf participation among older adults, specifically through the lens of the theory of planned behavior. By exploring the roles of event image, attitudes, subjective norms, and perceived behavioral control, this study confirms the significant influence of these variables on behavioral intention. Furthermore, social support is identified as a key moderating factor, particularly in strengthening the relationship between subjective norms and behavioral intention. The findings suggest that promoting positive event images, reinforcing social support, and fostering positive attitudes are important for encouraging sustained participation in park golf. This study offers both theoretical insights and practical strategies for improving participation rates, which ultimately contribute to the well-being and social connectedness of older adults.

## Figures and Tables

**Table 1:** Confirmatory factor analysis results.

Scale Items	Standardized Loadings	CR	AVE	Cronbach’s α
Event image				
Boring–Exciting	0.904	0.953	0.872	0.920
Sad–Joyful	0.865			
Gloomy–Cheerful	0.906			
Attitudes				
Playing park golf is extremely unpleasant–extremely pleasant.	0.846	0.939	0.837	0.914
Playing park golf is extremely unattractive–extremely attractive.	0.894			
Playing park golf is extremely worthless–extremely valuable.	0.907			
Subjective norms				
The people in my life (e.g., family/friends) would be in favor of me playing park golf.	0.824	0.945	0.852	0.905
The people in my life (e.g., family/friends) would support me playing park golf.	0.941			
The people in my life (e.g., family/friends) would encourage me to do well in park golf.	0.887			
Perceived behavior control				
It is entirely up to me whether I play park golf.	0.904	0.944	0.849	0.921
If I want, I can play park golf.	0.895			
I have enough knowledge and experience to decide whether to play park golf.	0.876			
Behavioral intention				
I will try to continue playing park golf.	0.923	0.949	0.861	0.939
I intend to continue playing park golf.	0.915			
I am willing to devote money and time to playing park golf.	0.914			
Social support				
The people around me are willing to help if I encounter any difficulties while playing park golf.	0.845	0.944	0.849	0.891
The people around me do not hesitate to praise me when I do well in playing park golf.	0.838			
The people around me show interest in my playing park golf.	0.891			

x²/*df* = 2.635; normalized fit index (NFI) = 0.942; relative fit index (RFI) = 0.926; incremental fit index (IFI) = 0.963; Tucker–Lewis index (TLI) = 0.953; comparative fit index (CFI) = 0.963, and root mean square error of approximation (RMSEA) = 0.074.

**Table 2:** Correlations between constructs.

Construct	Event Image	Attitudes	Subjective Norms	PBC	Behavioral intention	Social Support
Event image	0.934					
Attitudes	0.681 **	0.915				
Subjective norms	0.587 **	0.677 **	0.923			
PBC	0.637 **	0.690 **	0.674 **	0.921		
Behavioral intention	0.603 **	0.695 **	0.584 **	0.681 **	0.928	
Social support	0.484 **	0.403 **	0.590 **	0.414 **	0.371 **	0.944

** *p* < 0.01. Note: Diagonal elements in bold represent the square root of AVEs.

**Table 3:** Moderating effect of social support.

Estimation Type	Effect	Estimate	SE	*Z*	*p*
Moderation	Attitudes	0.875	0.041	21.15	<0.001
	Social support	0.107	0.049	2.24	0.025
	Attitudes × Social support	0.067	0.061	1.09	0.276
Moderation	Subjective norms	0.718	0.063	11.35	<0.001
	Social support	0.115	0.064	1.81	0.070
	Subjective norms × Social support	0.172	0.082	2.09	0.036
Simple slope	Average	0.718	0.064	11.30	<0.001
	Low (−1SD)	0.608	0.063	9.62	<0.001
	High (+1SD)	0.827	0.098	8.45	<0.001
Moderation	PBC	0.732	0.048	15.42	<0.001
	Social support	0.138	0.056	2.44	0.015
	PBC × Social support	−0.011	0.069	−0.16	0.871

## Data Availability

Data supporting the reported results can be made available by the corresponding author upon reasonable request.
